# The role of the cupola in the static and dynamic functioning of a lateral line stitch: natures non-linear application of Hooke’s law

**DOI:** 10.1080/19420889.2018.1542256

**Published:** 2018-11-21

**Authors:** Michelangelo Rossetto

**Affiliations:** Independent Scholar, NY, USA

**Keywords:** Lateral line, stitch, hair cell, Hooks law

## Abstract

The cupola of a lateral line stitch, once thought to simply be a large surface on which to collect vibrations, is now seen to be key for setting up a non-linear embodiment of Hooke’s law. By coupling, through the cupola, *oppositely* polarized hair cells in a primitive bilateral symmetry, a non-linear response dependence on input magnitude emerges. This non-linear response, which makes the transduction sensitivity a function of the mechanical input signal’s magnitude, engenders a new understanding of the vast, dynamic range of hair cells.

## Introduction

Fish can be understood as one large muscle with a life support and sensory system, along with sufficient intelligence to maintain their niche and adapt to the ongoing incremental mutations that improve their survivability. A creature’s survivability depends on its ability to be aware of its surroundings. In daylight, the eye is used to inform the fish about its surroundings. However, at night, the fish monitors its sound field just as humans listen in the dark to sense surrounding space acoustically.

**Figure 1.**
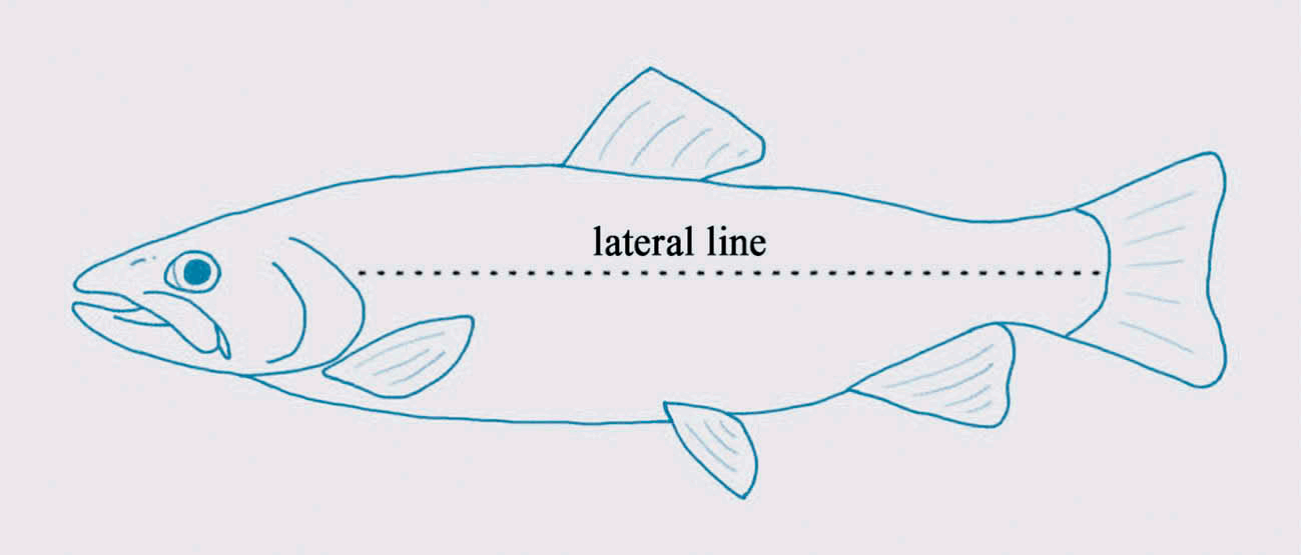
Image of a fish’s lateral line.

While humans use ears to keep track of the sounds around us, fish sense the surrounding space using its Lateral Line. The lateral line is a row of vibration sensors spaced along the side of the fish running from head to tail. These sensors, each composed of groups of vibration sensing hair cells, monitor vibrations in the water surrounding the fish. Each lateral line sensor, called a stitch, is represented by a dot in Figure 1

Each lateral line dot is an individual sensor that picks up a sound wave, or water born vibration, as it sweeps past. Each dot, the individual sensors, sends a fiber to the fish’s brain, where the relative arrival times of a vibration will be processed to determine the direction of arrival of the vibration as well as its characteristics.

If the direction of the vibration’s arrival is at a right angle to the body of the fish, all individual lateral line sensors will be stimulated simultaneously. However, if the vibration comes from a position near the front (head) of the fish, the sensors near the front are stimulated first. The stimulation then sweeps from the front to the back. Similarly, if the vibration comes from a position near the rear end of the fish, the stimulation sweeps the lateral line sensors from back to front. The lateral line, with its array of simultaneous detectors, provides a panoramic acoustic image of surrounding activity. This acoustic environmental sensitivity is available day and night, but in the night its importance to survivability emerges.

## Analysis of findings

**Figure 2.**
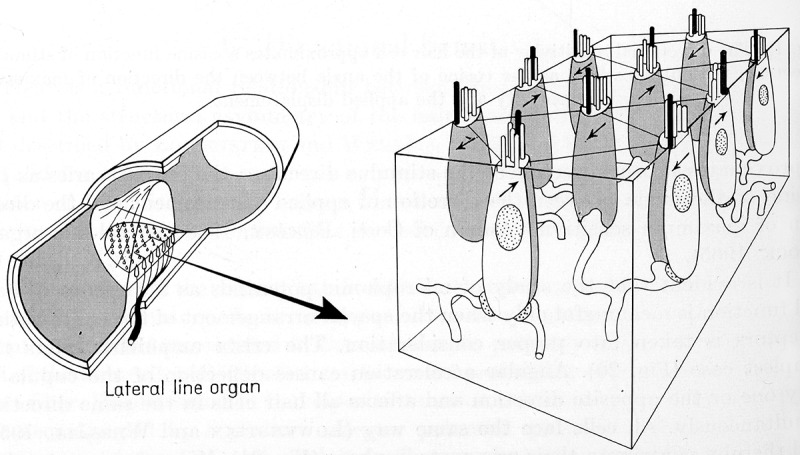
A schematic representation of the stitch organization from Flock [1].

Flock first addressed the description of the components, and their organization, of a lateral line stitch in 1962. He presented a drawing, reproduced below in Figure 2, that lays out the arrangement of the hair cells of the lateral line and how they support, and relate to, the cupola.

Flock’s drawing (Figure 2) shows how hair cells are arranged in oppositely polarized pairs. The arrows drawn on the hair cells show the polarity response. Deflection towards the kinocilium, the heavy dark line, is a positive stimulus. The kinocilium is longer than the cilia and inserts into the cupola. The cupola sits atop of the ensemble of hair cells, supported by the taller kinocilia. The input vibrations are transferred to the hair cells’ cilia through their kinocilia.

**Figure 3.**
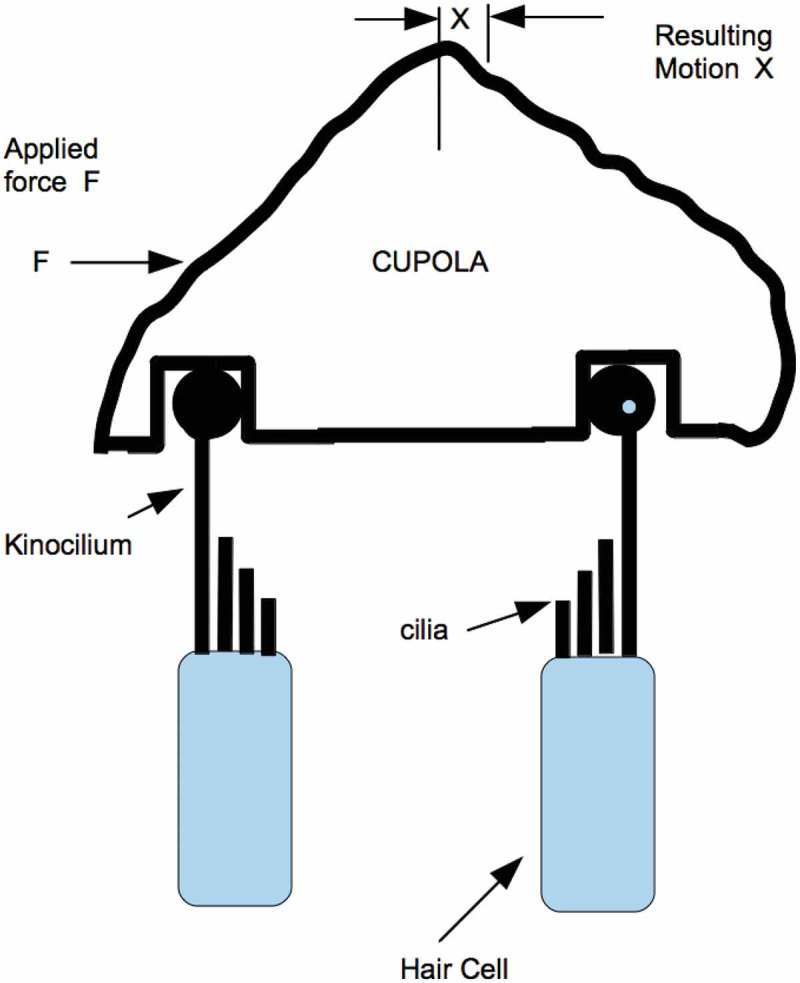
The cupola entrains the opposing fields of hair cells into a single system which expresses primitive bilateral symmetry.

A typical stitch will have around 25 pairs of opposing hair cells. The cupola constrains all pairs to act in concert. A single symbolic pair (as is shown in Figure 3 below) may, therefore, represent the entire stitch.

The cupola of a lateral line stitch has **two** distinct functions:

1. The cupola brings waterborne vibrations to the hair cells by catching aquatic vibrations with a surface area that is vastly larger than the area presented by hair cell cilia.
2. The cupola marshals together opposing polarity fields. Opposing hair cells push against each other through the cupola. The ‘push-push’ interaction of the hair cells across the cupola sets up the conditions underlying the dynamics of the entire stitch.

The kinocilia of opposing depolarized hair cells press against each other throughout the cupola. The thesis of this paper, that the lateral line stitch functions under a non-linear form of Hooke’s law, rests on this cupola behavior and three well-known concepts:

1) Hooke’s Law

(1) $$X=K_{1}F$$

or

(2) $$F=\frac{X}{K_{1}}$$

Where X is the displacement of an elastic body subject to a force F. K is the constant of proportionality, a signed numeric; it is a measure of stiffness, often called the spring constant.

2) – Hair cell cilia displacement toward the kinocilium proportionally depolarizes the cell [1]

- “For adapting OHCs, the receptor potential amplitude is a linear function of excitatory bundle displacement for amplitudes less than 50 nm” [2]

(3) $$V=K_{2}X$$

- Replacing X in equation 3 with equation 1 illustrates how the hair cell voltage depends on F [3].

(4) $$V=K_{2}K_{1}F$$

3) “In nearly all kinocilia cell voltage change induces active pointer-like deflection in a graded and tonic manner. [A voltage change of a given sign induces a deflection that counteracts mechanical stimuli producing a voltage change of the same sign”] [3]

(5) $$X_{K}=K_{3}V$$

We see that a force applied to the cupola results in a deflection of the cilia. Deflection of the cilia produces a voltage change. This voltage change elicits a deflection of the kinocilium. This deflection of the kinocilium can be seen as a force operating on an elastic body. The kinocilium is a deflectable beam that is acted on by a force generated within the kinociilum.

Since kinocilia of opposite polarity are arranged to oppose each other through the cupola (as shown in Figures 2 and 3) they cannot move in response to hair cell depolarization. The forces that would have bent the kinocilium are instead present as a static force or stress in each of the opposing kinocilia. The greater this force, the stiffer the integrated pair of kinocilia will appear to an applied external force. The coefficient in Hooke’s law (shown above) is no longer a constant; it becomes a function of the hair cell membrane voltage and consequently a function of the applied force.

(6) $$The\;new\;coefficient\;is\;a\;function\;of\;X\;:\;\;K^{1}\;X$$

(7) $$X=\frac{F}{K^{1}X}$$

(8) $$X^{2}=\frac{F}{K^{1}}$$

or

(9) $$\begin{array}{rl} & Taking\;the\;square\;root\;of\;both\;\\ & sides\;ofequation\;8:X=\sqrt{\frac{F}{K^{1}}} \end{array}$$

(10) $$\begin{array}{rl} & If\;for\;convenience\;we\;define\\ & \;a\;new\;constant:K=\sqrt{\frac{1}{K^{1}}} \end{array}$$

Equation (9) can be rewritten as;

(11) $$X=K\sqrt{F}$$

**Figure 4.**
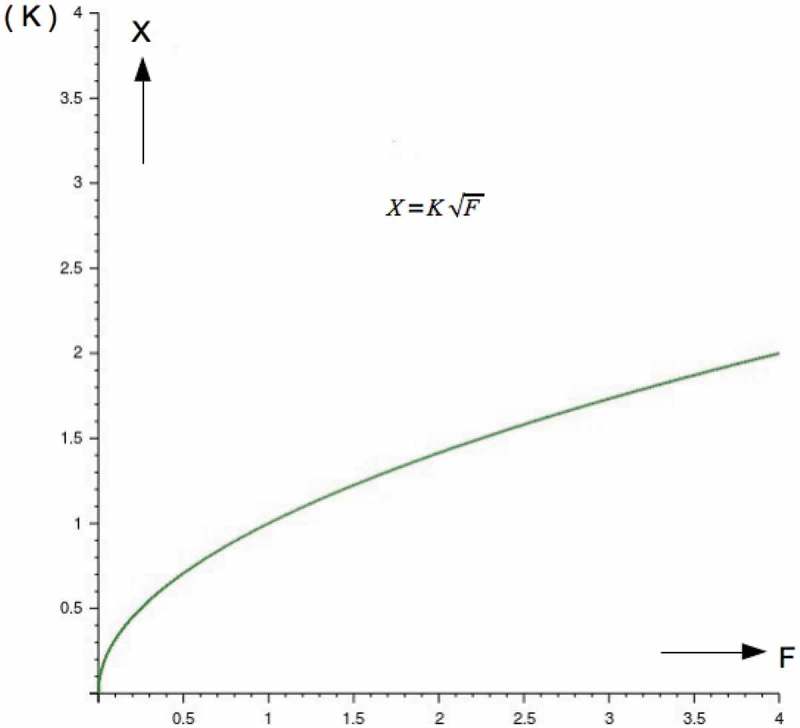
Illustrates the nonlinear response achieved by the hair cell, which allows it to avoid being overstimulated by large signals.

This is plotted in Figure 4:

The differential response to an applied force, shown, in Figure 4, diminishes as the magnitude of the force increases.

This is best visualized by taking the first derivative of $X=K\sqrt{F}$.

(12) $$\frac{dX}{dF}=\frac{K}{\left(2\sqrt{F}\right)}$$

**Figure 5.**
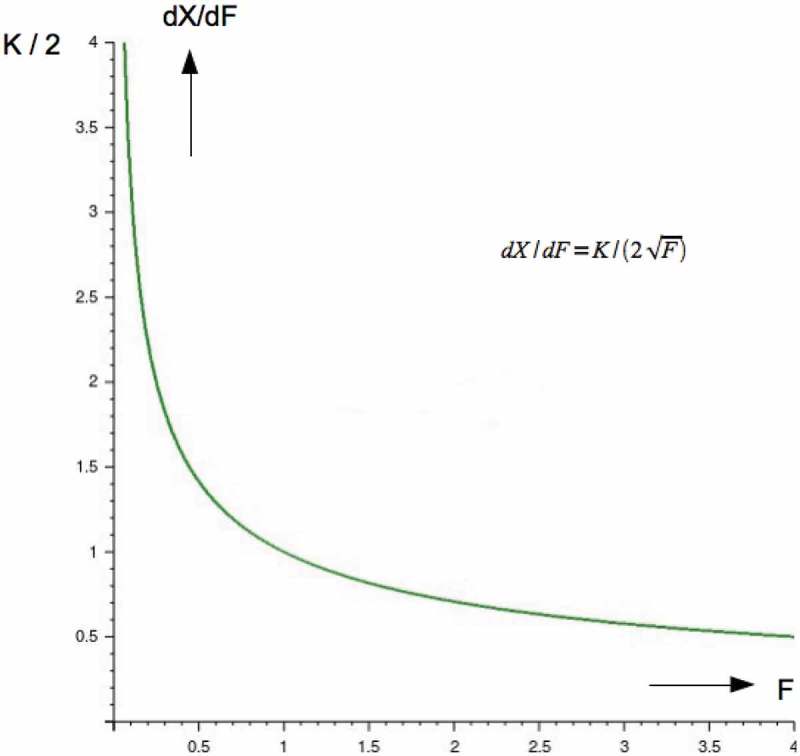
Shows $\frac{dX}{dF}=\frac{K}{\left(2\sqrt{F}\right)}$, a measure of transduction sensitivity as a function of applied force.

$\frac{dX}{dF}=\frac{K}{\left(2\sqrt{F}\right)}$ is plotted in Figure 5

Here we see how the sensitivity is a strong hyperbolic function. It becomes obvious that hair cells can cope with signals of varying sizes, from very small ones barely larger than the Brownian motion of the cilia to signals with increasing magnitudes.

## Conclusion

The extraordinary dynamic range of a lateral line stitch is set up by the cupola’s interaction with the opposing hair cells as shown in Figure 3. Functionally, this system stiffens as the signals to the lateral line increase and loosens and the applied signals get smaller. The opposing hair cells in Figure 3 are holding the cupola in a manner reminiscent of a basketball player holding the ball in preparation for a shot from center court. Both of these situations depend on bilateral symmetry. The lateral line stitch may be an example of an early application of bilateral symmetry. This primitive bilateral symmetry can also be found in the utricle and sacculus. In these organs, opposing polarized groups relate to each other across the striola, a dividing line on the sensory surface. In the utricle, they face each other across the striola. In the sacculus, they face away from the striola.

In the attempt to understand the functionality of the hair cell in sensory systems, effort is increasingly focused on finer and finer details of the hair cell’s structure and genetics, rather than viewing the system as a whole. This paper, hopefully, illustrates the value of a more *holistic* consideration of all available data.

We would be well advised to keep in mind the 1944 observation made by Anais Nin: “Too much lucidity creates a desert.” [4]
